# Supplementary figures and images for: CRMP/UNC-33 organizes microtubule bundles for KIF5-mediated mitochondrial distribution to axon
Source: PLoS Genet. 2021 Feb 11;17(2):e1009360. doi: 10.1371/journal.pgen.1009360 (PMC7904166; doi:10.1371/journal.pgen.1009360)

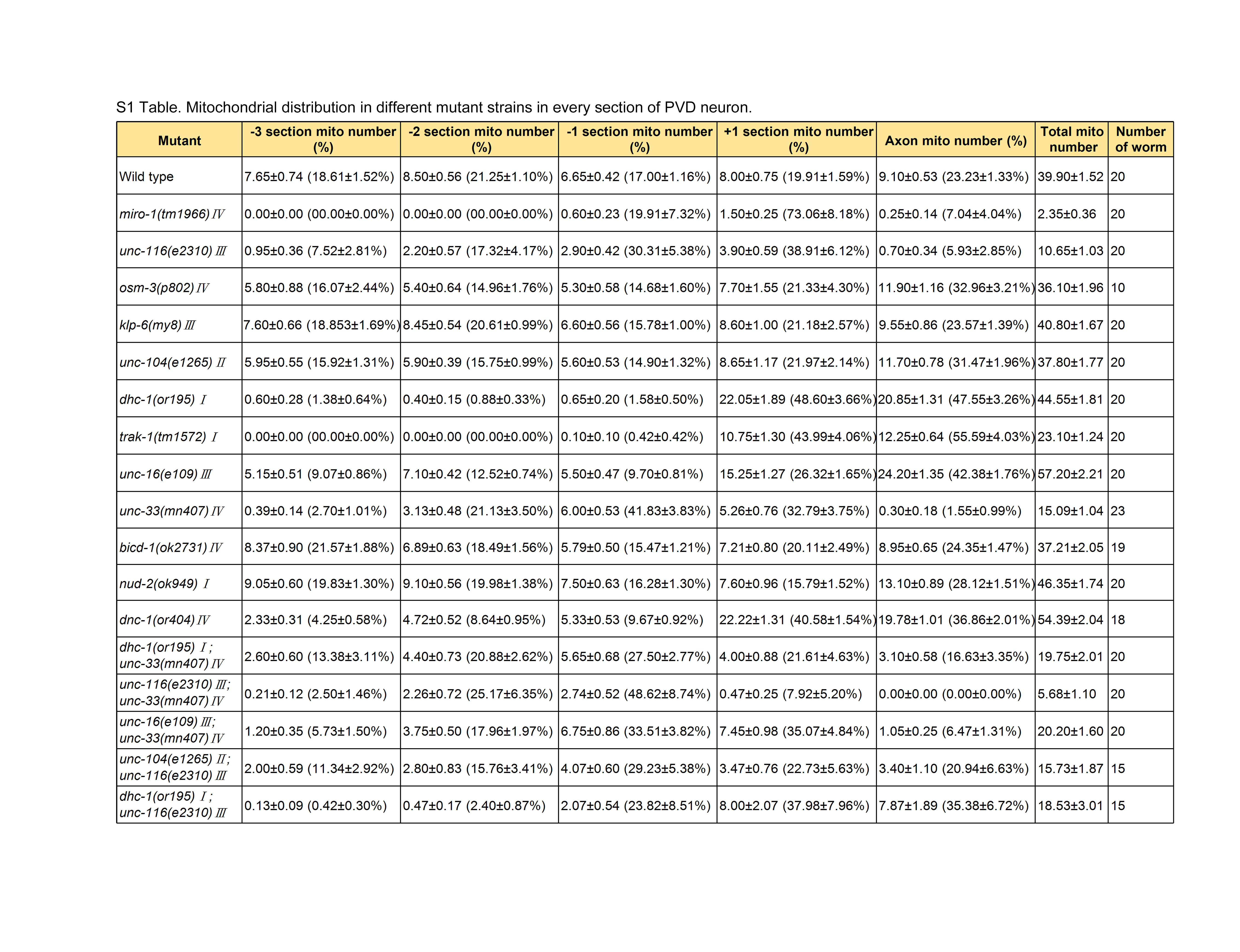

Supplement: S1 Table — (TIF) [file pgen.1009360.s005.tif]
