# Supplementary material for: CRMP/UNC-33 organizes microtubule bundles for KIF5-mediated mitochondrial distribution to axon
Source: PLoS Genet. 2021 Feb 11;17(2):e1009360. doi: 10.1371/journal.pgen.1009360 (PMC7904166; doi:10.1371/journal.pgen.1009360)
Supplement: Attachment — Submitted filename: Response to rerviewers.docx [file pgen.1009360.s009.docx]

Dear Editor,

We have revised our manuscript “CRMP/UNC-33 organizes microtubule bundles for KIF5-mediated mitochondrial distribution to axon” to address specific points made by reviewers. Below is the detailed list of our responses to reviewer’s comments with the description of changes in our manuscript.

Reviewer #1: The manuscript by Chan-Yen Ou group entitled “CRMP/UNC-33 organizes microtubule bundles for KIF5-mediated mitochondrial distribution to axon” evaluated how mitochondrial distribution in different compartments of neurons is regulated. They examined mitochondrial motors and transport regulators for their roles in mitochondrial transport using the PVD neuron as a model. This is an important topic, given that mitochondrial trafficking is essential for neuronal function and its dysregulation is involved in various neurodegenerative diseases.

Despite the characterization of factors regulating mitochondrial transport, how is this regulated in different cell compartments is less clear. The authors established a transgenic reporter for mitochondrial distribution in PVD neurons and examined how mitochondrial distribution was affected by loss of function of kinesins (unc-116, unc-104, klp-6 and osm-3), dynein (dhc-1), and transport regulators (trak-1, unc-16 and unc-33). They revealed distinctive roles of these genes in mitochondrial transport and analyzed how they genetically interact. As unc-33 is known to regulate microtubule organization, they went ahead to test whether unc-33 impact motor polarized mitochondrial distribution by regulating general cytoskeletal arrangement. They concluded that UNC-33 selectively stabilizes axonal microtubules to promote UNC-116-mediated mitochondrial transport to axons. Overall, I found the genetics experiments to be quite compelling. However, I believe a few issues will need to be addressed.

Major concerns:

1. The authors established a very nice genetic reporter to study mitochondrial transport in PVD neurons and identified specific roles of the motors and regulators in different compartments of the neuron. Are these findings neuron type-specific, or can these findings be applied to other neuron types, such as touch sensory neurons and DA9 neuron, which have long been used to study vesicular trafficking and microtubule organization?

As the reviewer suggested, we have generated several other genetic reporters in order to examine the role of UNC-33 in DA9 neuron, PDE neuron, and touch sensory neurons (ALM and PLM). While DA9 specific reporters showed no signals in *unc-33* mutant worms, we successfully examined mitochondrial distribution in touch receptor neurons and PDE neuron. In ALM, a main process projects anteriorly with a ventral branch to form synapses at the nerve ring. We found that mitochondrial number decreased to 30% in the main process in *unc-33* mutant, and mitochondrial distribution was almost abolished in the ventral synaptic branch (S2A and S2B Fig). Similarly, we also found similar phenotype in PLM neuron of *unc-33* mutant (personal observation, not shown). In PDE neuron, the axon grows ventrally and forms synapses to innervate ventral nerve cord. While the axon was shortened and the pathfinding was moderately defective, we found that PDE axonal mitochondria dramatically reduced in ventral synaptic region in *unc-33* mutant (S2E Fig). In summary, all these different neurons that we have examined showed dramatically reduced axonal mitochondria, especially in synaptic regions, in *unc-33* mutant.

We further examined microtubule organization by UNC-104-rigor::GFP and UNC-116-rigor::mCherry in ALM and PLM neurons. As PVD neuron, UNC-104-rigor and UNC-116-rigor label two distinct microtubule systems; UNC-104-rigor labels a mesh-like pattern surrounding the nucleus, and UNC-116-rigor labels clear microtubule bundles that form a spindle-like organization in cell bodies (left panels in S4 Fig). We observed that UNC-116-rigor labelled microtubule spindle-like organization disappeared in ALM and PLM cell bodies of *unc-33* mutant, while 104-rigor labelled microtubule remained relatively normal (right panels in S4 Fig). These results are consistent with the main idea of this work: while microtubules organize into different systems that recruit distinct motors, UNC-33 is specifically required to organize UNC-116-rigor-labelled microtubule network that supports axonal mitochondrial transport. These experiments on different neurons suggest that the role of UNC-33 in mitochondrial distribution and microtubule organization is conserved in different neurons.

2. The authors have concluded that “UNC-33 sticks MT bundles together to form a spindle-like high-order structure in the cell body and selectively maintains stabilized MTs for KIF5”. They did not provide images showing UNC-33 and UNC-116-rigor-GFP, but based on the Mossci reporter of UNC-33::GFP, the subcellular localization pattern of UNC-33 does not match with UNC-116-rigor-GFP pattern (i.e. spindle-like pattern in soma).

We agree with the reviewer’s comment that the UNC-33L::GFP pattern does not match with UNC-116-rigor::GFP pattern. Since the C-terminal region of CRMP1 is mapped to interact with microtubule (Lin et. al JBC 2011), the GFP domain at the C-terminal UNC-33L::GFP might interrupt microtubule interaction. Therefore, we inserted GFP after the globular domain at the 331 a.a. of UNC-33L to make UNC-33L::internalGFP according to a recent work (He et.al. eLife 2020), and co-injected it with UNC-116-rigor::mCherry. We found that UNC-33L::internalGFP formed a punctate pattern closely associated with microtubule bundles labelled by UNC-116-rigor::mCherry (Fig 5G). The pattern is consistent with the idea that UNC-33 interacts with microtubule bundles to promote their high-order organization.

3. They propose that UNC-33 adheres stable MT bundles to some structure of cell body cortex, which is likely to be the F-actin network beneath the cell membrane. Can they test this?

Yes, we expressed tagRFP::UtrCH to label F-actin network with UNC-33L::internalGFP. We found that UNC-33 puncta localize well with the F-actin bundles (Fig 5H). The localization of UNC-33 puncta are surrounding microtubule bundles but colocalize with F-actin beneath the cell membrane (brackets in Fig 5G and 5H).

4. They have shown that UNC-33 is localized in soma, axon and dendrites, then why UNC-33 selectively stabilize microtubules in axons? They discussed that this might not be due to microtubule polarity. Testing this in a different neuron type might help too.

Based on the observation of microtubule structure in ALM, PLM, and PVD neurons (S4 and Fig 5A), we found that UNC-33 is consistently required for high-order organization of microtubule bundles in these cell bodies. As the reviewer suggested in ALM neuron, while UNC-116-rigor signal was still present in the anterior process (S4A Fig), mitochondrial number severely decreased in this region in *unc-33* mutant (S2B and S2C Fig). These results agree with the idea that high-order microtubule organization in the cell body is required for mitochondrial distribution to neuronal processes.

5. Can microtubule targeting agents rescue the defects in unc-33 mutant?

We have tried to rescue the defects of *unc-33* by microtubule stabilizing agent paclitaxel but found that it is not sufficient to rescue the defects. As previously described (Chalfie M et. al. 1982 JCS, and Ritchie FK et. al. 2017 Cell Reports), we treated worms with 1 μM paclitaxel and observed no effect of axonal mitochondrial distribution in *unc-33* mutant, but we did observe numerous ectopic dendritic branches, possibly due to over-stabilized microtubules caused by paclitaxel. Therefore, we inferred that, although MT is stabilized by paclitaxel, UNC-33 is still needed to bundle microtubules and organize the high-order spindle-like structure for mitochondrial transport.

Minor issue:
The figures provided for review are in extremely low resolution, making it super hard to read and evaluate the data.

High resolution figures are available from online server. (There is a hyperlink in each figure on the pdf file)

Typo in line 342-343: “Whether dose CRMP/UNC-33 regulate mitochondrial transport by directly promoting kinesin-1 activity?”

We have corrected the error, now in line 358.

Reviewer #2: The manuscript by Ying-Chun Chen et al addressed how mitochondria is transported differentially in axon and dendrites using PVD neuron model in worm. They used mitochondrial reporter TOMM20::GFP and mutants deleting microtubule-based motors and their regulatory subunits to address the above-mentioned question. While it is really appreciated that PVD neuron would be a good system to look at the mitochondrial transport in neuron and authors develop a nice system to study this area. Using this system, the authors showed that KIF5/UNC-116 and dynein/DHC-1 is important for transport of mitochondria in axon and dendrites respectively. The mitochondrial adaptor Miro-1 plays an important role in mitochondrial distribution in all the neurites. According to the authors, more intriguing is the finding that CRMP family protein UNC-33 plays an important role in this process by regulating the organization of microtubule cytoskeleton in axon and dendrites. I completely agree that it is a nice characterization of this new system for studying mitochondrial transport. However, a major concern is that lack of new insight this study gives on mitochondrial distribution in neuronal cell.

1) For example, the role of KIF5/UNC-116 (Campbell et al 2014 J Neurosc, Han et al 2016 Neuron), Miro-1 (Han et al 2016 Neuron and many other studies) and dynein (van Spronsen M et al 2013 Neuron) is well established in axonal and dendritic transport of mitochondria. There are many other reports on the role of kinesin-1 and dynein in axonal transport of mitochondria.

The work of Campbell (Campbell et al 2014 J Neurosc) showed that KIF5A is important to transport mitochondrial to peripheral cutaneous axons in zebrafish, but little is known about the role of KIF5A in the dendritic region. The work of van Spronsen (van Spronsen M et al 2013 Neuron) characterized the role of TRAK1 and TRAK2 in cultured hippocampal neurons. Although these cultured neurons are convenient to study, they lose their original environment, and the development of their axon and dendrites is impaired due to the lack of guidance cues and synaptic targets. After all, it remains unclear how the distribution of mitochondria among different neuronal compartments in a native neuron is regulated by TRAK proteins in vivo.

In this work, we chose PVD neuron in worm because it has neuronal processes with distinct functional property and microtubule polarity including a MT minus-end-out dendrite, a MT plus-end-out dendrite, and a MT plus-end-out axon. Although many regulators like KIF5A and TRAK are known to regulate mitochondrial transport, this work reveals how these known regulators influence mitochondrial distribution among different subcellular compartments and evaluates their function in different neurite property (axon/dendrite) or in different MT polarity (plus-end-out/minus-end-out) in a consistent neuron. Therefore, it can provide us insights into how these regulators actually function in neurons.

For example, TRAK proteins potentially bind both kinesin and dynein as motor adaptors (van Spronsen M et al 2013 Neuron), but it is unclear whether they act differently in neuronal processes with different MT polarity (plus-end-out/minus-end-out) or with different structure property (axon/dendrite, synaptic/asynaptic, branch/main tract). In contrast, this work shows that *trak-1* mutant has nearly no mitochondria at the anterior dendrite (minus-end-out) but normal amount in posterior dendrite (plus-end-out) and the axon, while the total number of neurite mitochondria decreased 40% (Fig 2 and S1 table). Based on these data, this work shows that TRAK-1 is required for mitochondrial distribution in minus-end-out dendrite but not in plus-end-out dendrite and the axon, and it further shows that TRAK-1 has no differential activity between axon and dendrite when they have the same MT polarity (Fig 2F). These insights will help us to assess the role of TRAK-1 in a complete system.

Furthermore, this work examines other known motors in mitochondrial transport, and reveals their distinct roles in the same neuron instead of fragmented works in different cultured cells or in a restricted axonal region with different contradictory results. This work shows a complete mitochondrial distribution profile for each different regulator. Based on these profiles, researchers can evaluate the relative role of the regulator at different subcellular compartments and understand how a complete mitochondrial distribution system works.

2) The major finding that UNC-33 is important for mitochondrial transport lacks sufficient insight. For example, ‘Maniar et al 2011 Nat Neurosc’ clearly demonstrated that UNC-33 is important for establishment of microtubule polarity in axon and dendrites of PVD neuron. In the absence of the same MTs are disorganized and affects distribution of motors and cargoes in synapse and dendrites. Apart from that also other studies that highlight the importance of UNC-33/CRMP in neuronal microtubule organization in C. elegans (Martin Harterink et al 2018 JCS) and vertebrate model. Essentially, it’s important that authors highlight their findings that advances this field. On that basis, I feel that this study would be more suitable for elsewhere.

The work of Maniar et. al. (Maniar et. al. 2011 Nat Neurosc) showed that *unc-33* mutant has mis-localized synaptic vesicles and UNC-104/KIF1. However, ‘Maniar et al 2011 Nat Neurosc’ didn’t show how mitochondrial distribution is regulated by UNC-33. Furthermore, both ‘Maniar et al 2011 Nat Neurosc’ and ‘Harterink et al 2018 JCS’ showed microtubule polarity is mostly normal (with plus-end-out direction) in axon region. Here, in this work, we found that axonal mitochondria are almost completely abolished in *unc-33* mutant, and this phenotype can’t be explained by microtubule polarity. Furthermore, our study shows that the UNC-116-rigor and UNC-104-rigor associate with two different systems of microtubule organization, and UNC-33 is especially required for the formation of the spindle-like structure of UNC-116-rigor-associated microtubule in the cell body. The advance we made is that other than microtubule polarity, the construction of a special microtubule track is important to guide motor for mitochondrial transport to the right destination, such as the axon.

3) The authors may highlight how UNC-33 might be specifically important for mitochondrial transport? Can they provide some evidence by disturbing the specific function of UNC-33 related to mitochondria that only mitochondrial transport is affected without altering the distribution of other components such as RAB-3.

We found that the distribution of synaptic vesicles (RAB-3) is less affected by UNC-33 (around 33% of synaptic vesicle clusters are still present), which is in contrast to nearly no mitochondria present in axon in *unc-33* mutant (Fig 2C and 4F). The absence of UNC-33 causes moderate decrease (42%) of UNC-104-rigor-associated MT, but results in much severer decrease (80%) of UNC-116-rigor-associated MT. We concluded that, although it likely affects transport of cargoes in both dendritic and axonal compartments, UNC-33 is critical for axonal mitochondrial distribution.

4) The UNC-33L isoform was shown to regulate microtubule organization earlier (Maniar et al 2011). Although authors suggest M and S isoform does not rescue, no quantitative data is presented.

The quantification data of M and S isoforms is added in S3C Fig.

5) In figure-5, the authors claim that UNC-33 is involved in organization of UNC-116-bound microtubule tracks. How can they be sure that the other microtubule population is unaffected?

We indeed observed that UNC-104-rigor-associated MT signal was reduced in the cell body as mentioned above. However, we noticed that the strongest defects of *unc-33* mutant in microtubule structure is the loss of UNC-116-rigor-associated MT organization.

6) I suggest the authors that they proofread the manuscript for the language and grammatical errors. At times, it’s difficult to follow. It needs a major rewriting.

We have corrected errors according to comments from reviewers.

Reviewer #3: This work describes the roles of dynein and kinesin MT motor proteins, adapters, and the MT organizing molecule UNC-33/CRMP in mitochondrial distribution in neurons. The PVD sensory neuron has process with MTs of distinct polarities: + end out axon and posterior dendrite; and - end out anterior dendrite. They find that specific motor proteins and adapters are responsible for mitochondrial distribution in these processes. They also find that UNC-33/CRMP is required to establish a unique MT structure in the cell body utilized by UNC-116/KIF5 for mitochondrial entry into processes from the cell body. The mitochondrial distribution assay in the PVD neuron is nice, and is the basis of many experiments in the manuscript. The live imaging of mitochondrial entry into processes was good. These are interesting and significant results. The experiments are in general well designed and controlled, with appropriate statistical analyses. The figures, however, were of too low a resolution to assess some of the claims made by the authors in the text. My comments to improve the manuscript are below.

1) The resolution of the figures in the pdf copy that I downloaded was really low, making it difficult in some instances to see in micrographs what the authors describe in the text. Likewise, some of the graphs and data figures were small and at this resolution, difficult to see clearly.

Although we did upload the original high-resolution figures, the resolution of the pdf format automatically generated by the submitting system was low. Please notice that high-quality figures can be downloaded directly from the online server. (There is a hyperlink in each figure on the pdf file)

2) Many of the experiments rely on quantifying mitochondrial prevalence in distinct processes and sub-sections of processes. The authors make claims about increased or decreased mitochondrial movement. However, it is also possible that more or fewer overall mitochondria are being generated. This should be addressed in each experiment.

We have measured the total amount of mitochondria and added the data in S1 Fig. We found no significant change in *unc-33, unc-16, trak-1, nud-2,* and *dnc-1* mutants, and a small decrease in *unc-116* and *dhc-1* mutants. Since mitochondria in neurite decrease dramatically 73% in *unc-116* mutant, the major effect of UNC-116 is unlikely caused by the change of mitochondrial amount. Similarly, since *dhc-1* mutant showed 175% and 129% increases of mitochondria in posterior dendrite and axon respectively, and a severe 93% decrease in anterior dendrite, it is unlikely that this phenotype of *dhc-1* is caused by a small decrease of mitochondrial amount. Meanwhile, the distribution analysis in this work is based on the percentage of mitochondria in neurite or relative ratio between different segments, which is not affected by the total amount of mitochondria. Therefore, we conclude that total mitochondrial amount is unlikely the main factor for the distribution effects of these regulators.

3) Add a reference for the unc-33(mn407) deletion allele or add a figure with the deletion data.

We have added the reference of *mn407* in line 214, the number 33 reference.

4) The argument about unc-16/JIP3 gating rather than minus end specificity would be bolstered by analyzing total mitochondria in this mutant.

As above, no significant change of the total mitochondrial amount was observed in *unc-16* mutant.

5) The authors find that mutations in the dhc-3 dynein heavy chain partially suppress defects in unc-33 mutants. Because suppression is not complete, the authors argue that dhc-3 and unc-33 act independently and that unc-33 is unlikely to regulate mitochondrial transport via dhc-3. Is the suppression significant compared to unc-33 alone? If so, I would argue that this suggests that they do interact, and that dhc-3 might be one of multiple unc-33 partners in mitochondrial transport.

The loss of *dhc-1* can restore some axonal mitochondria caused by *unc-33* mutant (Fig 3E), and therefore, indeed, it is still possible that *dhc-1* acts partially downstream to *unc-33* (line 232-236).

6) Defects in the unc-33; unc-116 double mutant are increased relative to single mutants. Is this a synergistic effect, or additive? If it is synergistic (i.e. greater than the predicted additive effect of the mutations alone), this suggests parallel, converging pathways. If it is additive, this suggests mechanistically independent pathways. This should be addressed, as it gets at mechanism.

The total mitochondrial number was reduced 73% in *unc-116* and 62% in *unc-33* compared to wild type (Fig 3D). In *unc-116; unc-33* double mutant, total mitochondrial number was further reduced 62% compared to *unc-33,* and reduced 46% compared to *unc-116* (Fig 3B and Fig 3D). So, the results showed that the reduction in *unc-116; unc-33* double mutant is not greater than the predicted additive effect. Indeed, it suggested that UNC-33 might function in the parallel pathway in regulating mitochondrial distribution.

7) The authors mention that MT polarity is disrupted in some mutants. This seems like an important experiment to perform for all mutants, but especially for unc-33. Do unc-33 mutants have MT polarity defects?

Yes, *unc-33* mutants have MT polarity defect in the anterior dendrite, but mostly normal MT polarity in the posterior dendrite and the axon. According to ‘Harterink et al 2018 JCS’, they used EBP-2 to trace microtubule plus-ends. They found that the minus-end out polarity in the anterior dendrite is reversed to about 80% plus-end-out, while it remains plus-end-out in the posterior dendrite and the axon.

8) The Results section entitled “UNC-33 organizes microtubule bundles in PVD cell body and stabilizes microtubules in axon” is written in an unclear manner. Part of this is due to referring to MTs using distinct terminology. The authors switch between acetylated versus tyrosinated MTs, stable versus dynamic MTs, and those labeled by the unc-116rigor and unc104rigor. I would suggest described the reagents and what they selectively label, and then describe experiments by using the reagent name. For example, instead of
“In the absence of UNC-33, the spindle-like acetylated MT bundles were severely diminished in the cell body, and the general level of tyrosinated MTs in the cortical region of the cell body was also decreased, but the circular mesh of tyrosinated MTs was still present around the nucleus (Fig 5A and 5B).”
Use this terminology:
“In the absence of UNC-33, the spindle-like UNC-116rigor signal was severely diminished in the cell body, and the general level of UNC-104rigor signal in the cortical region of the cell body was also decreased, but the circular mesh of unc-104rigor was still present around the nucleus (Fig 5A and 5B).”

We have changed the terminology in description according to reviewer’s suggestion at line 287-290.

9) A reference for the rigor technique is missing.

The reference for rigor technique is added as reference 25 and 56 at line 536.

10) Given the resolution of the figures, it was impossible for me to see the spindle-shaped UNC-116rigor structure or the circular mesh of unc-104rigor in Figure 5. Because of this, it was impossible for me to assess the effect of unc-33 on these structures.

Please notice that high-quality figures can be downloaded directly from the online server. (There is a hyperlink in each figure on the pdf file)

11) Work from my lab has shown that unc-33 influences MT + end distribution in VD growth cones in vivo (Norris et al., 2014). The results presented here are consistent with this result. I would ask the authors to consider referencing this work in discussions about known roles of unc-33/CRMP.

We have added the work at line 347-348 and the reference 58.
